# Histone acetylation and methylation in rare diseases: from molecular mechanisms to clinical presentations

**DOI:** 10.3389/fcell.2026.1777625

**Published:** 2026-04-09

**Authors:** Burcu Akman, Semra Gürsoy, Pınar Gençpınar, Ayşe İpek Polat, Ayşe Semra Hız, Yavuz Oktay, Tahsin Stefan Barakat, Uğur Özbek, Serap Erkek-Ozhan

**Affiliations:** 1 Rare and Undiagnosed Diseases Group, İzmir Biomedicine and Genome Center, İzmir, Türkiye; 2 Department of Pediatric Genetics, Faculty of Medicine, Dokuz Eylül University, İzmir, Türkiye; 3 İzmir International Biomedicine and Genome Institute, Dokuz Eylül University, İzmir, Türkiye; 4 Department of Pediatric Neurology, Faculty of Medicine, Izmir Katip Celebi University, Izmir, Türkiye; 5 Department of Pediatric Neurology, Faculty of Medicine, Dokuz Eylül University, Izmir, Türkiye; 6 Department of Clinical Genetics, Erasmus MC University Medical Center, Rotterdam, Netherlands

**Keywords:** chromatin modifiers, chromatinopathies, epigenetics, neurodevelopment, rare disease

## Abstract

Rare diseases, which collectively affecting millions of people worldwide, present unique diagnostic and therapeutic challenges due to their low prevalence and phenotypic heterogeneity. The importance of epigenetic deregulations in the pathophysiology of rare diseases has been highlighted by recent research on neurodevelopmental diseases and congenital malformation syndromes. Among these, abnormalities in histone modifications (especially lysine methylation and acetylation) have emerged as one of the key mechanisms underlying disease phenotypes. Histone-modifying enzyme mutations result in a variety of developmental diseases, including Kabuki, Rubinstein-Taybi and Weaver syndromes, often manifesting as cognitive impairments, craniofacial abnormalities and growth deficiencies. This review explores the functional convergence of genes encoding histone modifiers and their roles in chromatin regulation. It also analyzes the distribution of variants in these genes and their association with overlapping phenotypes across rare diseases. The findings highlight how different variants within the same gene can result in diverse phenotypic outcomes, and how variants in distinct genes may manifest convergent phenotypes underscoring the interconnected nature of epigenetic deregulations and their implications for understanding genotype-phenotype relationships. By focusing on the subunits of key histone-modifying complexes, we also systematically mapped associated Mendelian phenotypes and highlighted a subset of genes not yet linked to defined syndromes but showing strong intolerance to loss-of-function variants, suggesting their potential involvement in undiagnosed or emerging neurodevelopmental disorders.

## 1 Introduction

**FIGURE 1:**
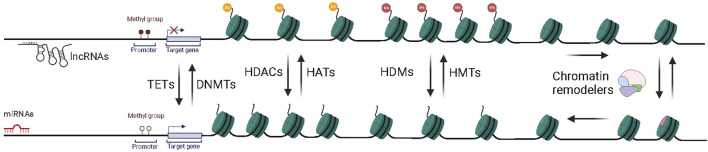
Key epigenetic mechanisms driving chromatin dynamics and transcriptional control. DNA methylation is catalyzed by DNA methyltransferases (DNMTs), typically leading to transcriptional repression. Histone modifications are mediated by histone acetyltransferases (HATs) and histone deacetylases (HDACs), which add or remove acetyl groups, respectively, to influence chromatin accessibility. Histone methylation dynamics are controlled by lysine methyltransferases (KMTs) and demethylases (KDMs), modulating gene activation or repression depending on the specific histone marks. Chromatin remodeling complexes reposition or evict nucleosomes to regulate access to DNA. Non-coding RNAs, including long non-coding RNAs (lncRNAs) and microRNAs (miRNAs), further contribute to gene regulation by guiding chromatin modifiers or mediating post-transcriptional silencing. Together, these mechanisms establish a dynamic and coordinated control of transcriptional activity.

Epigenetic mechanisms are critical to regulating gene expression and maintaining proper cellular processes, including cellular differentiation, development, and homeostasis (Zoghbi and Beaudet, 2016). DNA methylation, histone modifications, chromatin remodeling, and the regulatory functions of non-coding RNAs have unique roles as different layers of this regulation (Allis and Jenuwein, 2016) (Figure 1). Epigenetic perturbations that cause transcriptional aberrations are involved in disease pathogenesis and progression, such as cancers, neurological diseases, immunological and metabolic diseases (Dawson and Kouzarides, 2012; Jakovcevski and Akbarian, 2012; Surace and Hedrich, 2019; Tzika et al., 2018).

The growing interest in the role of post-translational modifications of histones, key determinants of chromatin state, in human disease has significantly advanced our understanding of the molecular mechanisms underlying various conditions and their clinical manifestations (Roberts et al., 2024). Among the epigenetic regulations, histone methylation, and acetylation have emerged as critical drivers of rare disease phenotypes (Bhat et al., 2021). These discoveries have highlighted the significance of epigenetics in rare disease pathogenesis and underscored the interplay between different epigenetic modifiers. Therefore, epigenetic dysregulations could be used as potential biomarkers for diagnosis and as potential targets for therapy.

In the United States, rare diseases are defined as conditions affecting fewer than 200,000 individuals, while in the European Union, they are typically defined as affecting fewer than 1 in 2,000 individuals (Boycott et al., 2017). If considered at a collective level, rare diseases are not as rare in the general population, affecting approximately 6%–8% of the global population (Ferreira, 2019). In fact, some of these diseases are ‘rare’ in some populations, but not in others. There are an estimated 7,000 different rare diseases affecting more than 350 million people globally. More than 70% of rare disease have a genetic origin (Groft et al., 2021; Haendel et al., 2020). Despite advances in molecular and bioinformatic techniques, there is no effective treatment or management strategy for nearly 90% of rare diseases.

This review highlights the role of histone modifiers in rare disease pathogenesis, covers the clinical manifestations associated with these diseases, and discusses emerging therapeutic approaches targeting the epigenetic landscape. By discussing these aspects, we aim to provide a comprehensive understanding of how histone modifications drive disease and offer insights into current challenges and potential opportunities in rare disease research.

## 2 Function of histone modifiers in gene regulation

Histone proteins are essential components of nucleosome structures and are subjected to post-translational modifications (PTMs), such as methylation, acetylation, phosphorylation, and ubiquitylation. Histone PTMs are regulated by specific enzymes that catalyze, recognize, or remove modifications. These modifications can shape chromatin configuration by changing chromatin accessibility and interactions between nucleosome complexes. The level of chromatin condensation orchestrates DNA accessibility for specific transcription factors, chromatin remodeling complexes, and other DNA/chromatin binding factors, (Bannister and Kouzarides, 2011; Peterson and Laniel, 2004) together ensuring correct spatiotemporal gene expression. Chromatin states are significantly associated with the establishment and maintenance of cellular identity, progression of cellular differentiation (Allis and Jenuwein, 2016).

Histone lysine methylation and acetylation, which are the focus of this review, are common histone marks. These marks are modulated in a highly dynamic way by histone methyltransferases (KMTs) and histone acetyltransferases (HATs), together known as “writers”, as well as histone demethylases (KDMs) and histone deacetylases (HDACs), often referred to as “erasers” (Bannister and Kouzarides, 2011; Berger, 2002). Allowing a more open chromatin structure, histone acetylation is generally linked to active transcription. On the other hand, depending on the specific residues methylated (such as H3K4, H3K9, H3K27) and the extent of methylation, histone methylation can either contribute to transcriptional repression or activation. The chromatin state can also be altered by chromatin remodeling complexes, such as the SWI/SNF complex, which regulates accessibility and nucleosome localization (Sudarsanam and Winston, 2000). The diverse functions of chromatin remodeling complexes, and interplay between different histone modifications, add an extra level of complexity to the regulation of gene expression during embryonic development and tissue-specific differentiation.

To control the transcriptional machinery and regulate stable cell fate during development, some complexes, such as Polycomb group (PcG) and Trithorax group (TrxG) proteins, function as antagonists. TrxG proteins promote gene expression by depositing H3K4me3 and by facilitating chromatin activity. In contrast, Polycomb Repressive Complex 1 (PRC1) contributes monoubiquitylation of H2AK119 and chromatin compaction, while Polycomb Repressive Complex 2 (PRC2) catalyzes the trimethylation of H3K27 via its catalytic component EZH2. Together, they maintain epigenetic memory and enable dynamic switches in gene expression throughout developmental processes (Yao et al., 2016). These two protein complexes are critical for the establishment and resolution of bivalent chromatin states. Bivalent state that is especially observed in embryonic stem cells, carries both activating (H3K4me3) and repressing (H3K27me3) histone marks. This phenomenon occurs at the promoters of key genes for developmental processes. In the presence of bivalent state, promoters can be rapidly switched on or off based on the underlying cellular differentiation program (Bernstein et al., 2006; Mas et al., 2018). Recent studies demonstrated that chromatin bivalency is not only a mechanism for embryonic stem cells but also a universal mechanism in tissue-specific gene regulation. This mechanism can be misregulated as a result of increased cellular plasticity in many diseases (Glancy et al., 2024).

Taken together, perturbations in this highly coordinated balance between histone writers, erasers and chromatin remodeling complexes through either environmental influences or germline or *de novo* variants affecting genes encoding epigenetic regulators cause profound biological consequences. Indeed, an increasing number of rare neurodevelopmental disorders have been linked to pathogenic variants in genes encoding chromatin-regulating proteins. Recently, these disorders have been referred to as chromatinopathies. A detailed understanding of the molecular functions, enzymatic activities, domain architecture and interaction networks of histone modifiers is critical for elucidating disease mechanisms, improving gene–disease associations and exploring potential therapeutic strategies.

## 3 Clinical manifestations of rare diseases associated with histone modifiers and convergence of functional mechanisms

Mutations in chromatin-modifying genes result in rare neurodevelopmental diseases with wide-ranging clinical variability (Supplementary Table 1). Although these rare diseases arise from mutations in distinct chromatin-modifying genes, they frequently display shared phenotypes, reflecting the central role of chromatin regulation in transcriptional programs during embryogenesis.

**FIGURE 2:**
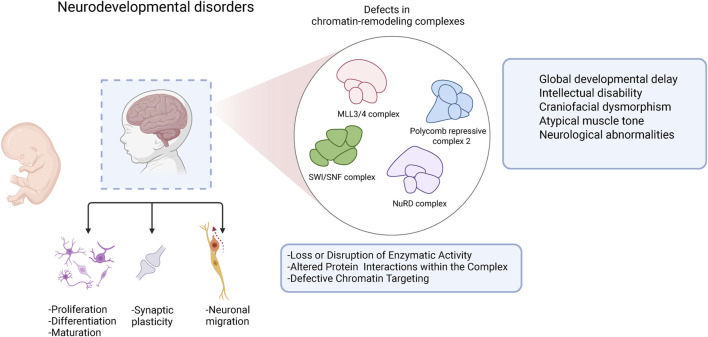
Impact of chromatin modifier defects on neurodevelopmental disease mechanisms.Loss-of-function or gain-of-function mutations in chromatin remodeling complexes can lead to altered chromatin states, dysregulated gene expression, and impaired lineage specification. These molecular disruptions affect neuronal differentiation, migration, synaptic development, and plasticity. The cumulative effects can result in a spectrum of neurodevelopmental disorders, including intellectual disability, developmental delay, and facial dysmorphism.

During neurodevelopmental stages, histone-modifying and chromatin remodeling complexes (e.g., MLL, NuRD, SWI/SNF, PRC2) regulate critical processes including neurogenesis, neuronal migration and synaptic plasticity. Genetic alterations affecting components of these complexes can perturb chromatin dynamics through reduced enzymatic activity, disrupted protein–protein interactions within the complex, or aberrant chromatin targeting (Fahrner and Bjornsson, 2019). Such disruptions lead to widespread transcriptional misregulation and give rise to a broad spectrum of overlapping clinical manifestations, including global developmental delay, intellectual disability, craniofacial dysmorphisms and neurological abnormalities (Bjornsson, 2015) (Figure 2).

Among the diseases summarized in Supplementary Table 1, here we specifically focus on Kabuki (MIM: 147920; 300867), Kleefstra (MIM: 610253), *KMT2C*-related (MIM: 617768), Weaver (MIM: 277590), Rubinstein-Taybi (MIM: 180849; 613684), *HDAC4*-related (MIM: 619797) and Cornelia de Lange (MIM: 300882) syndromes because they represent well-established monogenic neurodevelopmental disorders directly caused by variants in core histone modifier genes. These focused conditions collectively span key chromatin regulatory axes governing histone methylation, acetylation and chromatin accessibility during development. By concentrating on these prototypical chromatinopathies, we aim to illustrate how disruption of distinct yet interconnected epigenetic mechanisms converges on aberrant developmental gene regulation.

**FIGURE 3:**
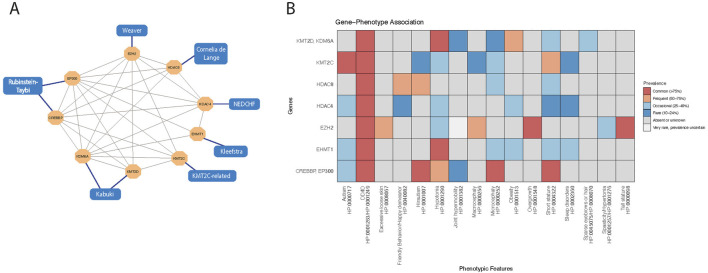
Gene–phenotype associations in rare neurodevelopmental disorders involving chromatin-modifying genes. This figure illustrates the relationships between chromatin modifier genes and their associated neurodevelopmental phenotypes using two complementary visualization strategies. **(A)** A gene–disease interaction network showing chromatin regulator genes (hexagonal nodes) and associated clinical syndromes (rectangular nodes). Gene–gene interactions were inferred from the STRING database and visualized using Cytoscape. Edges between genes represent predicted or experimentally supported molecular interactions, while edges connecting genes to phenotypes reflect curated gene–disease associations derived from the literature. **(B)** A heatmap summarizing gene-phenotype correlations in rare neurodevelopmental disorders involving histone-modifying genes. Rows represent individual genes, and columns denote commonly observed clinical phenotypes. Color intensity indicates the frequency of each phenotype associated with each gene, categorized as Common (>75%), Frequent (50%–75%), Occasional (25%–49%), Rare (10%–24%), Very rare (prevalence uncertain), or Absent/Unknown. This visualization highlights both converging and gene-specific clinical features, enabling comparative phenotypic profiling across epigenetic disorders. Phenotype prevalence was inferred from peer-reviewed literature, including case series and cohort studies (Frazier et al., 2025; Imagawa et al., 2023; Kaiser et al., 2014; Le et al., 2019; Murakami et al., 2020; Pérez-Grijalba et al., 2019; Rots et al., 2024b). The heatmap was generated using the ggplot2 package in RStudio (version 2024.04.2 + 764). This visualization highlights both converging and gene-specific clinical features, enabling comparative phenotypic profiling across epigenetic disorders.

The molecular and clinical relationships among these disorders have been illustrated in Figure 3. Implicated genes form a densely interconnected chromatin regulatory network rather than representing isolated functional units (Figure 3A). Many of these proteins participate in shared or functionally convergent complexes that coordinate histone methylation, acetylation and chromatin accessibility during development. Consistently, this molecular interdependence is mirrored at the phenotypic level. Gene-phenotype mapping (Figure 3B) reveals a marked overlap in core neurodevelopmental features, particularly developmental delay (DD), intellectual disability (ID), characteristic craniofacial features and hypotonia. This parallel convergence at both the molecular and phenotypic levels supports the concept that disruption of interconnected chromatin regulatory mechanisms underlies the shared clinical spectrum observed across these chromatinopathies.

**FIGURE 4:**
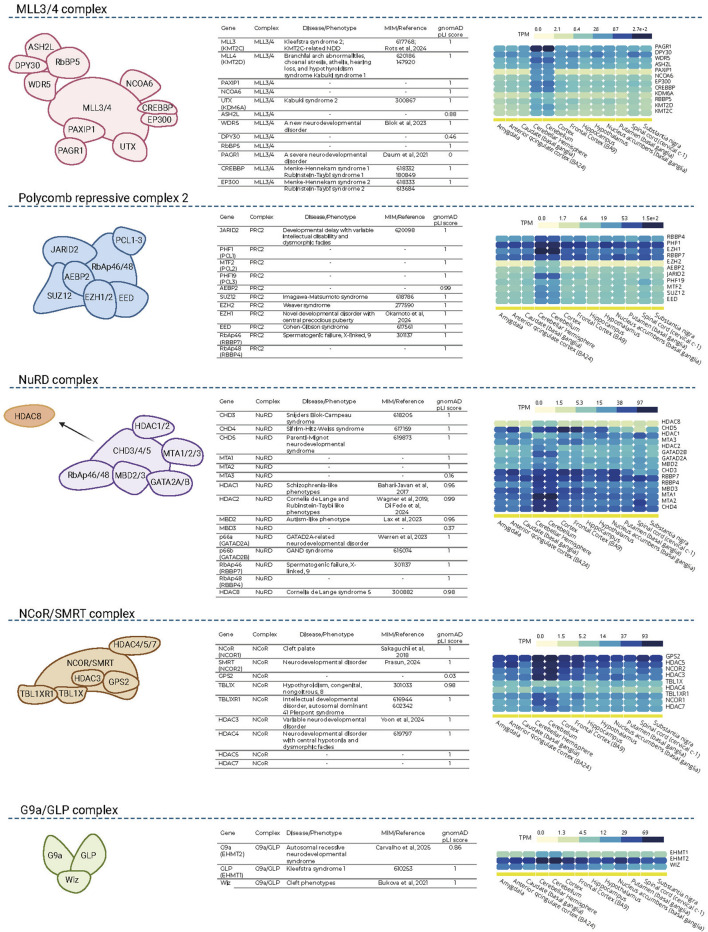
Systematic mapping of histone-modifying complexes: gene content, expression dynamics, and clinical relevance. The figure is organized into five subsections corresponding to the MLL3/4, PRC2, NuRD, NCoR/SMRT, and G9a/GLP complexes. Within each subsection, a complex schematic is shown on the left, a gene-level summary table is provided in the middle, and GTEx-derived brain expression heatmaps (TPM) are displayed on the right. The tables list known Mendelian phenotypes with corresponding MIM numbers (based on OMIM data) and, where no OMIM phenotype is defined, literature-supported disease associations (Bahari-Javan et al., 2017; Bukova et al., 2021; Carvalho et al., 2025; Daum et al., 2022; Di Fede et al., 2024; Lax et al., 2023; Okamoto et al., 2024; Prasun, 2024; Rots et al., 2024b; Sakaguchi et al., 2018; Snijders Blok et al., 2023; Wagner et al., 2019; Werren et al., 2023; Yoon et al., 2024). Gene-level intolerance to loss-of-function variation is indicated by gnomAD pLI scores (v4.1.0; where unavailable, v2.1.1 values are reported). Brain-specific expression patterns across multiple anatomically distinct regions are visualized as GTEx-derived transcript per million (TPM) heatmaps (right panels), enabling comparison of tissue-level expression within each complex. Together, this integrative framework highlights the convergence of complex membership, genetic constraint, and neuroanatomical expression patterns in prioritizing candidate genes for rare neurodevelopmental disorders.

In line with this, our integrative analysis maps the disease relevance of core components across five major histone modifying complexes (PRC2, MLL3/4, NCoR, NuRD and G9a/GLP) (Figure 4). A substantial fraction of subunits are already implicated in well-defined Mendelian phenotypes underscoring the vulnerability of chromatin regulatory hubs to genetic perturbation. Notably, several additional complex members display high pLI scores in gnomAD, indicating strong intolerance to loss-of-function variation suggesting that they represent strong candidates for yet-unrecognized chromatinopathies. This genetic constraint aligns with consistent expression across multiple brain regions, reinforcing their developmental relevance (GTEx-derived profiles in Figure 4). Taken together, these findings highlight that chromatinopathies are best understood as disorders of interconnected epigenetic systems rather than isolated gene defects. Recognizing this network-level vulnerability may help refine how we prioritize candidate genes and interpret variants in rare neurodevelopmental disease (Figure 4).

### 3.1 Histone methylation and rare diseases

The proteins encoded by *KMT2D* (MIM: 602113), *KDM6A* (MIM: 300128), *KMT2C* (MIM: 606833), *EHMT1* (MIM: 607001) and *EZH2* (MIM: 601573) are histone methylation-regulating genes that have opposing roles on chromatin dynamics and gene expression. KMT2D, KMT2C and KDM6A are components of the MLL3/4 complex. KMT2D and KMT2C are H3K4 methyltransferases catalyzing mono-methylation, while KDM6A is a H3K27 demethylase removing repressive mark H3K27me3. It has been shown that regulation of these chromatin-modifying activities is critical for early development and lineage specification (Herz et al., 2012; Lee et al., 2013). Studies on knockout (KO) mouse models have demonstrated severe differentiation defects at the neural progenitor cell (NPC) stage (Xie et al., 2023). In contrast, EHMT1 and EZH2 act as transcriptional repressors as a part of distinct chromatin regulatory complexes. While EHMT1 catalyzes repressive H3K9me1 and H3K9me2 marks interacting with the NuRD and PRC2 complexes (Marques et al., 2020; Mozzetta et al., 2014), EZH2 serves as the catalytic subunit of PRC2 complex, depositing H3K27me3 (Margueron and Reinberg, 2011). Both of these results in chromatin condensation and gene silencing. Additionally, EHMT1 forms a functional heterodimer with EHMT2 (G9a) within the G9a/GLP complex, which mediates widespread H3K9 dimethylation and contributes to transcriptional repression during neural development (Benevento et al., 2016; Tachibana et al., 2008). As demonstrated in a recent study, the histone methyltransferase activity of EHMT1 plays crucial roles in neural development, neural network formation and synaptic plasticity, neural stem cell proliferation and differentiation, and it is also involved in learning and memory processes (Koemans et al., 2017). Furthermore, PRC2 proteins have been involved in development balancing self-renewal and differentiation of stem and progenitor cells (Schuettengruber et al., 2017). It has also been shown that these complexes play roles in controlling the dynamics of gene expression patterns during cortex development (Albert and Huttner, 2018; Bölicke and Albert, 2022).

Neurodevelopmental diseases linked with pathogenic variants in *KMT2D*, *KDM6A*, *KMT2C* and *EHMT1* exhibit phenotypic overlap (Figure 3B; Supplementary Table 1). Affected individuals frequently exhibit dysmorphic facial features, growth retardation, mild to moderate intellectual disability, hypotonia, seizures and behavioral abnormalities, as well as a spectrum of neurological and gastrointestinal manifestations.

#### 3.1.1 Kabuki syndrome

**FIGURE 5:**
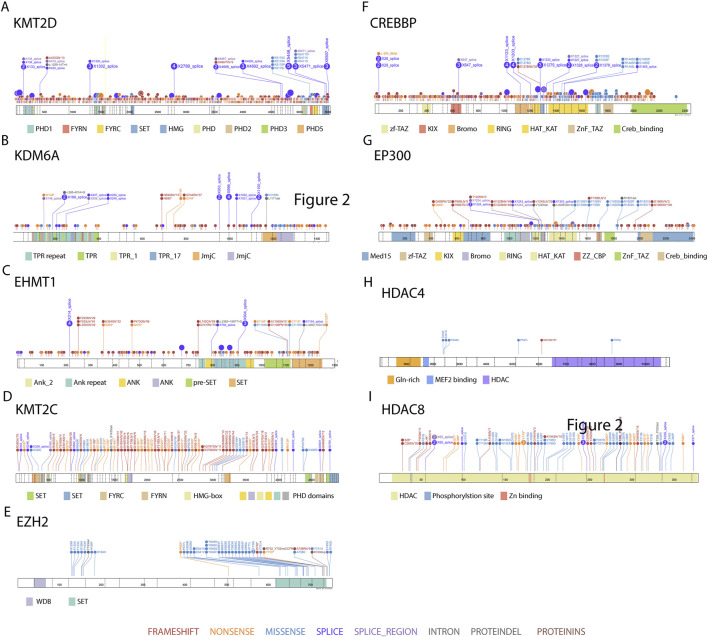
Distribution of clinically reported variants in chromatin modifier genes implicated in neurodevelopmental diseases. Lollipop plots summarize the distribution of clinically reported variants across selected chromatin modifier genes implicated in neurodevelopmental diseases (**(A–I)**: KMT2D, KDM6A, EHMT1, KMT2C, EZH2, CREBBP, EP300, HDAC4, HDAC8). Variant data were retrieved from ClinVar, and only entries classified as pathogenic or likely pathogenic were included. Visualizations were generated using ProteinPaint (release version 2.95.0), and data were accessed on 10 January 2025.

Mutations in *KMT2D* (also known *as MLL4*) were first identified and represent the main genetic cause of Kabuki syndrome, covering approximately 56%–75% of cases (Kabuki Syndrome 1). Subsequently *KDM6A* was discovered as related with Kabuki syndrome (Banka et al., 2012; Lederer et al., 2012; Miyake et al., 2013) and is mutated in less than 5% of affected individuals (Kabuki Syndrome 2). Although several mutational hotspots have been identified, pathogenic variants are distributed throughout the entire coding regions of both genes (Figures 5A,B).

At molecular level, KMT2D and KDM6A function within the MLL3/4 complex, where KMT2D catalyzes enhancer associated H3K4me and KDM6A removes the repressive mark H3K27me3 (Herz et al., 2012; Lee et al., 2013). Loss-of-function variants in both genes are therefore predicted to impair coordinated enhancer activation and disrupt the balance between activating and repressive chromatin states during development.

Clinically, Kabuki syndrome is characterized by distinct craniofacial features such as long palpebral fissures, arched and sparse eyebrows, cleft lip/palate, a depressed nasal tip and prominent ears. Skeletal findings often include short stature, joint laxity or dislocations, scoliosis and brachydactyly (Barry et al., 2022; Kuroki et al., 1981; Lintas and Persico, 2018). Phenotypic features such as congenital heart defects and short stature might differ in prevalence between Kabuki syndrome type 1 and type 2 (Adam et al., 2019). However, these differences may vary across different cohorts of Kabuki syndrome type 1 and type 2 as well. Additionally, a recent genotype-phenotype study employing AI-based approaches has demonstrated that facial morphology can distinguish between the two types, indicating consistent facial feature differences (Hennocq et al., 2024).

Studies in animal models have demonstrated that chromatin modifier genes implicated in Kabuki syndrome play essential roles in neural crest development. In mouse models, *Kdm6a* has been shown to be required for neural crest cell formation and migration, as well as for proper anterior cranial bone development (Shpargel et al., 2017). In *Xenopus*, loss of *Kmt2d* results in defects in neural plate border establishment and neural crest specification (Schwenty-Lara et al., 2020). These findings align with the classification of Kabuki syndrome as a neurocristopathy.

#### 3.1.2 Kleefstra syndrome

Kleefstra syndrome (KLEFS1) is caused by microdeletions or single nucleotide variants in *EHMT1* gene. Inspection of the *EHMT1* mutations associated with KLEFS1 reveals that the variants are distributed across the SET, ankyrin repeat and RING-like domains, as well as the N-terminal region of the protein (Figure 5C). DNA methylation signature analyses have shown that some variants within the SET domain and N-terminal region may not exhibit the characteristic Kleefstra syndrome methylation signature even they are associated with a clinical phenotype consistent with classical Kleefstra syndrome. This suggests potential variability in the epigenetic consequences of these mutations. Consistent with the role of *EHMT1* in depositing repressive H3K9me2 marks within the G9a/GLP complex, pathogenic variants are predicted to impair heterochromatin organization and destabilize transcriptional repression during neurodevelopment. Such disruption may lead to inappropriate activation of developmentally regulated gene programs, particularly in neuronal lineages, thereby contributing to the core neurodevelopmental phenotype observed in Kleefstra syndrome.

A characteristic phenotype of Kleefstra syndrome is its distinctive craniofacial phenotype, such as brachycephaly, broad forehead, flat midface, broad nasal bridge, hypertelorism, thick eyebrows and an everted lower lip. This facial phenotype serves as a key diagnostic feature. In addition to the distinguished facial gestalt, individuals with Kleefstra syndrome generally present with epilepsy, congenital cardiac anomalies, renal and urinary tract abnormalities, recurrent upper respiratory infections and hearing impairment. Recent studies have also indicated a high prevalence of sleep disturbances in affected individuals (Frazier et al., 2025; Rots et al., 2024a).

#### 3.1.3 *KMT2C*-related disorder

*KMT2C*-related disorder (previously classified as Kleefstra Syndrome 2) is caused by mutations in *KMT2C*. A recent study characterizing the clinical phenotype and epigenomic signatures of *KMT2C*-related patients has revealed that individuals previously classified under the term “Kleefstra Syndrome 2” exhibit distinct neurodevelopmental and molecular profiles than Kleefstra Syndrome 1. According to those findings, the *KMT2C*-associated disorder is now considered a separate neurodevelopmental condition (Rots et al., 2024b). While Kleefstra and Kabuki syndromes are clinically well recognized and often diagnosed through a phenotype-first approach, the facial gestalt in *KMT2C*-related patients was initially recognized as a distinct clinical entity. These patients were described as *EHMT1*-negative patients with Kleefstra-like phenotype. However, PhenoScore-based analysis demonstrated that this disorder exhibits a statistically distinct and consistent facial pattern, differentiating it from Kleefstra and Kabuki Syndrome 1. In terms of inheritance, *KMT2C*-related neurodevelopmental disorder also diverges from these syndromes: whereas Kleefstra and Kabuki 1 syndromes are predominantly caused by *de novo* loss-of-function variants, approximately 15% of *KMT2C* cases involve inherited variants from similarly affected parents (Rots et al., 2024b). The distribution of reported *KMT2C* variants is shown in Figure 5D, which spans all over the protein without a major hotspot. As a core catalytic component of the MLL3/4 complex, KMT2C contributes to enhancer-associated H3K4 mono-methylation and activation of developmental gene programs (see Section 3.1). Pathogenic variants are therefore expected to impair enhancer function and disrupt transcriptional regulation during neurodevelopment. The clinical distinction from Kabuki and Kleefstra syndromes may reflect differential sensitivity of specific enhancer networks to perturbation of individual MLL3/4 subunits.

#### 3.1.4 Weaver syndrome

Pathogenic variants in *EZH2* cause Weaver syndrome, an overgrowth syndrome largely distinct from other histone modifier-associated rare diseases (Figure 3B). A large portion of *EZH2* variants implicated in Weaver syndrome affect the catalytic SET domain (Figure 5E). As the catalytic subunit of PRC2, EZH2 is responsible for depositing the repressive H3K27me3 mark, which constrains developmental gene expression programs and limits cellular proliferation (see Section 3.1). Pathogenic variants affecting the SET domain are therefore predicted to impair PRC2-mediated repression, potentially leading to derepression of growth-promoting and lineage-specific gene networks. This epigenetic imbalance provides a plausible molecular basis for the overgrowth phenotype characteristic of Weaver syndrome.

Clinically, this disruption manifests as a pre- and postnatal overgrowth syndrome, a distinctive facial appearance (including hypertelorism and retrognathia) and variable degrees of intellectual disability. Tall stature is observed in approximately 90% of affected individuals, while intellectual disability, often mild, affects around 80% of them. Other clinical findings may include soft, doughy skin, a low hoarse cry, umbilical hernia and camptodactyly. Early manifestations can be detectable in prenatal period, such as excessive fetal growth on ultrasound, and macrocephaly and increased body size relative to age in neonatal period (Weaver et al., 1974). Consistent with the role of PRC2 in constraining proliferation, impaired EZH2 activity may also predispose to tumorigenesis, which aligns with the increased incidence of pediatric tumors observed in affected individuals (Connolly et al., 2024).

### 3.2 Histone acetylation and rare diseases

Mutations in the histone acetyltransferases or deacetylases such as *CREBBP* (MIM: 600140)*, EP300* (MIM: 602700)*, HDAC4* (MIM: 605314) and *HDAC8* (MIM: 300269), have been implicated in a spectrum of rare neurodevelopmental syndromes. Lysine acetyltransferases (KATs) and transcriptional co-activators, CREBBP (also known as CBP or KAT3A) and EP300 (also known as P300 or KAT3B), regulate gene expression in a highly tissue-specific manner (Lipinski et al., 2019). Even though they are not core components of the MLL3/4 complex, CREBBP and EP300 interact with chromatin remodeling complexes to maintain an active chromatin state (Figure 3A). Through their intrinsic KAT activity, they promote histone acetylation, enhancer activation and open chromatin configuration, facilitating transcriptional permissiveness during development, particularly in neurogenesis and cell fate determination (Goodman and Smolik, 2000; Heintzman et al., 2007; Rada-Iglesias et al., 2012).

Both CREBBP and EP300 are highly expressed in brain regions (such as the hippocampus and prefrontal cortex) that are important for learning and memory (Van Gils et al., 2024). In addition to early neuronal development, including the differentiation of motoneurons, cortical progenitors and astrocytes (Valor et al., 2013), they contribute to postnatal neuroplasticity and memory formation (González-Martínez et al., 2022; Lopez-Atalaya et al., 2011). At the molecular level, ab KATs have been associated with reduced promoter binding and decreased histone acetylation at key neuronal and glial gene loci (Wang et al., 2010). These underlie cognitive and behavioral impairments in related diseases.

Likewise, histone deacetylase (HDAC) family genes, such as *HDAC4* and *HDAC8*, play pivotal roles in neurodevelopment. These proteins cooperate with epigenetic corepressor complexes, like NCoR (SMRT), CoREST and NuRD (Bantscheff et al., 2011), and are essential for neural commitment and stem cell lineage decisions (Podobinska et al., 2017). HDAC4 is highly expressed in the brain and plays role in neuronal development, synaptic plasticity and long-term memory. Notably, both its overexpression and deficiency have been shown to impair cognitive function, suggesting a critical need for HDAC4 homeostasis in brain physiology (Wu et al., 2016). Actually, HDAC4 itself lacks intrinsic deacetylase activity. However, it can influence histone deacetylation through its catalytically active interactors including other HDACs (Lee et al., 2015). HDAC8 has been demonstrated to have key roles in neuronal differentiation during early development, including embryoid body formation (Morii and Inazu, 2022). Taken together, the intricate balance between histone acetylation and deacetylation governed by these enzymes is crucial for both early neurodevelopmental processes and adult cognitive plasticity.

*CREBBP/EP300*, *HDAC4* and *HDAC8*-related syndromes show overlapping phenotypic features, even with their distinct genetic origins and enzymatic activity. Intellectual disability, developmental delay and short stature are common to all three, with variable severity (Figure 3B). Characteristic facial dysmorphisms are present across all syndromes, even differ in specifics. Behavioral abnormalities and multi-system involvement (including cardiac, gastrointestinal and sensory systems) are also common. The functional convergence of *CREBBP/EP300*, *HDAC4* and *HDAC8* in modulating neurodevelopmental gene programs may underlie these phenotypic similarities, highlighting the critical role of chromatin dynamics in early brain development.

#### 3.2.1 Rubinstein-Taybi syndrome

Rubinstein-Taybi Syndrome (RSTS) is caused by pathogenic variants in *CREBBP* and *EP300*. RSTS, is caused by pathogenic variants in two paralogous lysine acetyltransferases (sharing approximately 61% sequence similarity across their functional domains) that function as transcriptional coactivators (Dancy and Cole, 2015). RSTS is inherited in an autosomal dominant manner, occurring in approximately one in 100,000 to 125,000 live births (Hennekam, 2006; Hennekam et al., 2005).

At molecular level, CREBBP and EP300 mediate histone acetylation at promoters and enhancers. Loss-of-function variants are therefore expected to reduce histone acetylation and impair enhancer activity, leading to diminished transcriptional output of key neuronal and developmental gene networks. Such global coactivator insufficiency likely underlies the multisystem and cognitive phenotype observed in RSTS. Figures 5F,G illustrate that pathogenic variants are broadly distributed across multiple functional domains of CREBBP and EP300, including the KAT domain, bromodomain, TAZ, and zinc finger motifs.

The lack of domain-specific enrichment further supports the notion that the global integrity of these multifunctional coactivators is critical for their role in development and neurocognitive function. Clinically, RSTS is characterized by craniofacial features including microcephaly, highly arched eyebrows, downslanting palpebral fissures, highly arched palate, low-hanging columella, convex nasal ridge and grimacing smile. Skeletal findings such as angulated and broad thumbs or halluces are the other cardinal abnormalities. The growth is generally normal in the prenatal period. However, growth deficiency starts in the first year of life. *CREBBP* variants account for 55%–75% of RSTS cases, while *EP300* variants are observed in approximately 10%, including 2%–3% of individuals with full gene deletions (Cohen et al., 2020; Roelfsema et al., 2005).

#### 3.2.2 *HDAC4*-related syndrome

Mutations in *HDAC4* underlie *HDAC4*-related neurodevelopmental disorder, a rare condition also known as neurodevelopmental disorder with central hypotonia and dysmorphic facies (NEDCHF) (Wakeling et al., 2021) (Figure 5H). Currently reported number of pathogenic variants remains limited, and those are hypothesized to disrupt its normal cellular localization and function. Normally HDAC4 is isolated by 14-3-3 proteins in the cytoplasm, which prevents its entry into the nucleus (McKinsey et al., 2000). However, mutations that impair this process may lead to increased levels of nuclear HDAC4, where it can aberrantly suppress the expression of its targets involved in neurodevelopment and tissue differentiation (Haberland et al., 2009; McKinsey et al., 2000; Wakeling et al., 2021). Dysregulation in its functions can lead to a wide spectrum of developmental and neurological syndromes. While neurodevelopmental features such as developmental delay and hypotonia are generally shared within multiple *HDAC*-related disorders, individuals with this syndrome also exhibit distinctive craniofacial features, such as hypertelorism, long palpebral fissures, a full lower lip and widely spaced teeth, as well as skeletal findings like joint hypermobility and scoliosis. These phenotypic characteristics may help in the diagnosis of this syndrome distinguishing from other histone deacetylase-related ones in clinical practice (Wakeling et al., 2021).

#### 3.2.3 Cornelia de Lange syndrome

Cornelia de Lange Syndrome (CdLS) is associated with pathogenic variants in genes encoding components of the cohesin complex and its regulators. To date, eight genes have been identified the CdLS syndrome, including *NIPBL* (MIM: 608667), *SMC1A* (MIM: 300040), *SMC3* (MIM: 606062), *RAD21* (MIM: 606462), *HDAC8* (MIM: 300269), *BRD4* (MIM: 608749), *ANKRD11* (MIM: 611192) and *MAU2* (614560) (Kline et al., 2018; 2007). *NIPBL* is the most frequently mutated gene, accounting for approximately 60%–80% of molecularly diagnosed patients and plays a central role in cohesin loading and transcriptional regulation.

Among these genes, *HDAC8*, occupies a unique position at the intersection of cohesin biology and chromatin regulation (Lucia-Campos et al., 2022). In addition to its role in cohesin maintenance through deacetylation of SMC3, HDAC8 modulates chromatin structure by deacetylating histones and cohesin-associated proteins (Mordaunt and McLauchlan, 2015). Through this activity, HDAC8 influences chromatin accessibility and enhancer–promoter communication, thereby contributing to transcriptional regulation during neural differentiation and cortical development. Disease-causing variants in *HDAC8* are distributed throughout the gene without clustering on any specific domain (Figure 5I). The lack of a defined mutational hotspot highlights the importance of *HDAC8* overall structural and catalytic integrity for proper neurodevelopment.

Abnormal HDAC8 activity during neurodevelopment contributes to the intellectual disability and behavioral manifestations observed in CdLS individuals carrying *HDAC8* mutations (Deardorff et al., 2012). The prominent manifestations of male patients with *HDAC8* variants include dysmorphic facial features that overlap with CdLS, but they are more often characterized by ocular hypertelorism, telecanthus, wide nose, delayed closure of anterior fontanelle, happy personalities, small hands and feet (Kaiser et al., 2014; Kline et al., 2018; Lucia-Campos et al., 2022). The X-linked inheritance of *HDAC8* further distinguishes this subgroup from other CdLS spectrums.

## 4 Current therapy options

**FIGURE 6:**
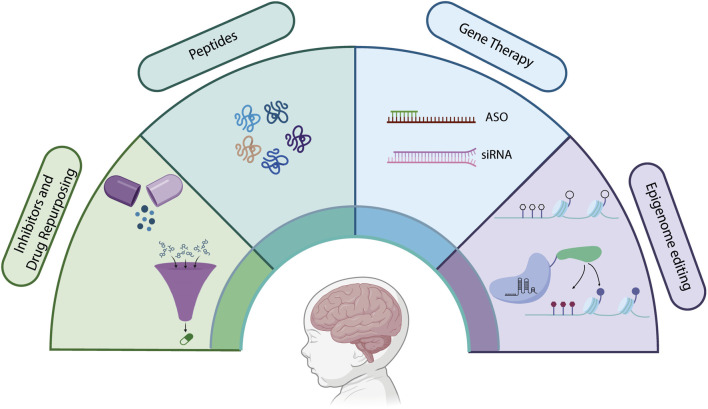
Emerging therapeutic strategies for neurodevelopmental diseases. Approaches include: (1) small-molecule inhibitors of histone modifiers and drug repurposing efforts aimed at restoring epigenetic balance; (2) synthetic peptides that mimic lost or dysfunctional histone modifications to re-establish chromatin signaling; (3) cell and gene therapies designed to replace or correct the function of affected genes or cell populations; and (4) epigenome editing technologies that allow locus-specific modulation of chromatin states using tools such as CRISPR-dCas9-based epigenetic effectors. These innovative strategies represent promising avenues for correcting gene regulatory disruptions underlying neurodevelopmental pathology.

As rare diseases often start early and in the prenatal period, prompt treatment is highly crucial. However, even after successful diagnosis, which is far from routine, for only 5% of rare diseases a treatment is available and only about half of therapy development efforts against rare diseases focus on non-oncological RDs (Chen et al., 2024). Moreover, most treatments alleviate symptoms rather than curing the disease. A main challenge in therapy development against most rare developmental disorders is the irreversible nature of defects caused by the disease-causing pathogenic variants. In this regard, disorders caused by mutations in histone modifiers represent a unique therapeutic scenario: although the genetic defect is permanent, the downstream chromatin state remains potentially modifiable. Thus, the dynamic and reversible nature of epigenetic modifications makes them attractive targets for therapeutic applications. Particularly, histone modification processes hold significant promise as therapeutic targets for treating a diverse range of diseases, from neurogenetic diseases to cancers. Indeed, a significant fraction of cancer drug development efforts focuses on small molecule inhibitors to target histone and chromatin-modifying enzymes to restore the normal epigenetic state, with several epigenetic drugs undergoing different clinical phases or being already approved (Dai et al., 2024). However, the therapeutic logic established in oncology does not directly translate to histone modifier–related rare diseases: whereas cancer therapies often suppress hyperactive pathways, many chromatinopathies arise from germline loss-of-function or haploinsufficient mutations, making direct enzyme inhibition mechanistically counterintuitive. Still, despite promising advances, there remains significant challenges in developing effective therapies for rare diseases associated with histone modifiers. One of the main obstacles is the lack of specificity for most drugs targeting epigenetic processes, which results in off-target and unintended effects on the regulation of gene expression, particularly given that histone modifiers regulate thousands of genomic loci in a cell-type–specific manner Moreover, overcoming the blood-brain barrier is another major obstacle for neurodevelopmental diseases. Additional limitations include incomplete understanding of disease-relevant transcriptional networks, uncertainty regarding therapeutic windows and the potential need for long-term epigenetic reprogramming rather than transient pathway modulation. Therefore, in addition to small molecule inhibitors of histone modifier enzymes, alternative therapy approaches that target the epigenetic processes are being increasingly explored, recently (Figure 6).

### 4.1 Inhibitors of histone modifiers and drug repurposing

Epigenetic therapies based on KDM inhibitors (KDMis), KMTis, HATis and HDACis are constantly being updated, however, a limited number of them are FDA-approved. Most currently available compounds originate from oncology-driven discovery pipelines, which partly explain their predominant inhibitory mode of action. According to the World Health Organization’s (WHO) International Clinical Trials (ICTRP) database, in 51 studies HDAC targeting molecules -by itself or combinatorially-were tested or being tested as rare disease treatment, while a search with the keyword “HAT” yields 55 results, “KMT” yields 0, and lysine demethylase yields 4 (https://trialsearch.who.int/, accessed 19 April 2025). While these HAT inhibitors find wider use against cancers, due to their enzyme-inhibiting activities, they are usually not ideal for rare diseases caused by inherited loss-of-function mutations in epigenetic regulator genes. Interestingly, in the cell lines from RSTS patients with reduced HAT activity, decreased histone acetylation levels were reversed upon HDAC inhibitor treatment (Lopez-Atalaya et al., 2012). These findings illustrate an important principle in chromatinopathies: therapeutic benefit may arise not from restoring the defective enzyme itself, but from rebalancing opposing epigenetic activities within the chromatin regulatory network. Additionally, some cognitive impairments were rescued via HDAC inhibitor treatment in RSTS mouse models (Lopez-Atalaya et al., 2012).

However, as exemplified by the use of HDAC inhibitors in preclinical models, their potential benefits via inhibition of overactive pathways in patients with rare diseases remain to be explored.

Nevertheless, HDAC inhibitors also exemplify key limitations, including broad transcriptional effects, limited molecular specificity and potential toxicity associated with long-term administration, particularly in pediatric patients. Furthermore, their effects are often transient, raising questions regarding treatment durability and optimal dosing strategies. Future progress will likely depend on the development of context-selective modulators, reader-domain inhibitors or allosteric regulators capable of fine-tuning chromatin states rather than globally inhibiting enzymatic activity.

### 4.2 Peptides that mimic the lost histone modifications

Beyond traditional small-molecule drugs, recent drug discovery efforts have explored and developed advanced therapeutic options (Liu et al., 2023). While still at early stages of development, a promising approach is to design peptides that mimic post-translationally modified histones, such as acetylated lysine residues. The principle of this strategy is to provide binding targets for chromatin readers and thereby modulating regulation of gene expression. This strategy may be particularly relevant for disorders in which disrupted recruitment of chromatin reader proteins, rather than catalytic failure alone, represents the primary pathogenic mechanism. However, major challenges remain, including intracellular delivery, nuclear localization efficiency, peptide stability and the combinatorial nature of histone marks, which may limit the efficacy of single-modification mimetics.

With the recent breakthroughs in *de novo* protein design with the help of large language models (LLM)-based AI tools, significant advances could be expected in this line of research. AI-assisted protein design may enable multi-domain chromatin interactors capable of reproducing complex epigenetic signaling environments, although this approach remains largely preclinical.

### 4.3 Gene therapies

While protein-based therapeutics are used for protein replacement and antigen neutralization, oligonucleotide therapies, particularly RNA-based therapies allow a wider range of options to target epigenetic processes (Bubenik et al., 2024). Among the most widely used RNA-based therapies, small interfering RNAs (siRNAs) and antisense oligonucleotides (ASOs) promote the mRNA degradation, modulate splicing, alter translation efficiency or interfere with miRNA binding. Their adaptability enables development of variant-specific or allele-selective approaches, which are particularly relevant for gain of function or dominant negative mutations in chromatin modifying genes.

While several RNA-based therapies have been approved and are in use against neuromuscular disorders, such as DMD (Ferlini et al., 2021; Goemans et al., 2018), their use with regard to histone modifiers have recently gained more attention. Indeed, the physical isolation of the nervous system from the rest of the body means ASOs and other treatment modalities that benefit from targeted administration can achieve high concentrations in the nervous system with minimal spreading to other tissues/organs. This therapy option is tightly linked to effective delivery strategies that overcome the blood–brain barrier (BBB). Because systemically administered RNA-based therapeutics often fail to cross the BBB. Intrathecal delivery, direct administration into the cerebrospinal fluid, allows high local concentrations of ASOs or other RNA modalities within the central nervous system while minimizing systemic exposure. Recent clinical successes of ASO-based therapies have demonstrated the feasibility and safety of this approach (Rinaldi and Wood, 2018). For a comprehensive review on potential applications of ASOs against *KMT2*-associated neurogenetic diseases, please see (Zardetto et al., 2024).

However, translating RNA-based therapies to histone modifier–related rare diseases presents unique challenges. Many of these conditions arise from haploinsufficiency of dosage-sensitive chromatin regulators, where both insufficient and excessive gene activity can be pathogenic. Therapeutic modulation must therefore achieve precise quantitative restoration rather than simple overexpression or suppression. Moreover, histone modifiers function as global regulators of gene expression; partial correction at the transcript level may have widespread downstream transcriptional consequences that are difficult to predict. The timing of intervention is also critical, as many chromatinopathies originate during early neurodevelopment, potentially limiting the reversibility of established epigenetic and transcriptional programs.

RNA-based therapies are part of a larger group of therapies, named “advanced therapy medicinal products” (ATMPs), which encompass gene therapies, somatic cell therapies and tissue-engineered therapies by the European Medicines Agency (EMA). Compared to ASOs, a more recently developed technology, mRNA therapy, has immense potential, as evidenced by the SARS-CoV-2 pandemic. Although mRNA therapies hold promise for transient restoration of functional protein, their use in chromatinopathies will require careful consideration of dosage control, cell-type specificity and long-term epigenetic stability.

### 4.4 Epigenome editing

Among gene therapy strategies, genome and epigenome editing has gained particular attention due to its specificity, flexibility and the expanding toolbox offered by modification of the CRISPR/Cas system. With successful proof-of-principle type studies that aim to increase the expression of wild-type allele (for haploinsufficient cases) or silence the mutant allele (dominant-negative cases) reported in cellular models of neurogenetic diseases (e.g., Kleefstra syndrome, Weaver syndrome), epigenome editing is a rapidly growing area of research in rare disease field (McCutcheon et al., 2024). One recent clinical trial (NCT03855631) explored the therapeutic potential of epigenome editing, where patient primary cells were reprogrammed into mesenchymal stem cells and edited by CRISPR/Cas9 system to develop an *in vitro* model of Kabuki Syndrome (Jovic et al., 2022).

Beyond CRISPR-mediated single base editing which enables the correction of disease-associated variants to restore the function of chromatin-modifying genes, recent advances in epigenome editing have made it possible to directly target and achieve specific epigenetic modification at specific genomic regions. This emerging strategy allows gene regulation without altering the DNA sequence. By fusing a catalytically inactive Cas9 (dCas9) with various epigenetic effector domains, such as histone acetyltransferases/methyltransferase or DNA methyltransferases, researchers can modulate the chromatin state at specific loci (Ansari et al., 2022) (Figure 6).

Despite its conceptual appeal, the application of epigenome editing to chromatinopathies presents important biological and technical challenges. Many of these disorders result from disruptions in global epigenetic regulators that control extensive enhancer networks, suggesting that correction of a limited number of loci may not fully restore complex transcriptional programs. The stability of engineered epigenetic changes and the risk of unintended chromatin remodeling remain key considerations, particularly in developing tissues. In addition, because many histone modifier–associated conditions originate early in neurodevelopment, postnatal intervention may not completely reverse established structural and circuit-level abnormalities. Therefore, successful clinical translation will require precise targeting strategies, careful safety assessment and a more deeper understanding of network-level epigenetic regulation.

## 5 Challenges and opportunities in rare disease research

Rare neurodevelopmental diseases associated with chromatin modifiers are particularly challenging because of context-dependent and dynamic mechanisms. These characteristics complicate diagnosis and research. Intra-syndromic heterogeneity, overlapping features across different syndromes and limited functional validation of variants also contribute to these obstacles. To overcome these challenges, approaches that consider the unique biology and diagnostic difficulties of epigenetic neurodevelopmental disorders are needed.

Phenotypic manifestations may depend on developmental timing, tissue specificity and other environmental factors particularly relevant for epigenetic regulation. Lack of well-defined patient cohorts remains a bottleneck for both translational and functional studies, which is slowing down discovery of therapeutic options. However, the development of global data-sharing initiatives and public resources such as OMIM (http://omim.org), gnomAD (https://gnomad.broadinstitute.org/), ClinVar (http://www.ncbi.nlm.nih.gov/clinvar/) and DECIPHER (https://www.deciphergenomics.org/) has greatly improved variant interpretation and provided broader data harmonization across studies (Amberger et al., 2015; Firth et al., 2009; Gudmundsson et al., 2022; Hamosh, 2004; Landrum et al., 2014). These platforms are highly valuable for epigenetic neurodevelopmental disorders, where functional annotation is often limited. The recent creation of resources like the “Rareservoir” database, which has linked hundreds of rare conditions to novel genetic drivers by analyzing whole-genome data from over 77,000 individuals, underscores the power of large-scale efforts to uncover hidden etiologies (Greene et al., 2023). In addition, tools like Matchmaker Exchange (https://www.matchmakerexchange.org/) and GeneMatcher (http://www.genematcher.org) facilitate phenotype–genotype matching for ultra-rare or newly described genes, including chromatin regulators, thereby supporting gene discovery and diagnosis in this challenging subgroup (Philippakis et al., 2015; Sobreira et al., 2015).

Still, a major limitation persists: many variants in chromatin-related genes remain classified as variants of uncertain significance (VUS), due to a lack of functional evidence. Emerging multi-omics tools not only offer new and improved approaches for identifying disease-causing variants but also enable the development of downstream functional assays that assist in interpreting VUS. As sequencing capacity expands and the volume of detected variants increases, the integration of such complementary functional readouts into diagnostic pipelines is becoming essential to fully realize the clinical value of genomic data (Kernohan and Boycott, 2024). Another emerging opportunity lies in rethinking about gene discovery and variant prioritization from the perspective of protein complex biology. Traditional approaches often focus on individual genes and their direct genotype–phenotype associations. Chromatin-modifying enzymes, on the other hand, rarely act in isolation; instead they function as part of multiprotein complexes in a well-coordinated way. Viewing undiagnosed neurodevelopmental phenotypes through the architecture of known chromatin complexes may therefore reveal overlooked candidate genes (Figure 4). For example, when a causal variant is found in a subunit of chromatin-modifying complex, its interacting partners (especially those with high intolerance to disruptive variation) should be flagged for clinical re-evaluation in genetically unsolved cases. This network-informed perspective not only better captures the functional organization of chromatin biology but also enables us to formulate of mechanistically grounded hypotheses about disease etiology, even in the absence of clearly defined phenotypic associations. Systematically leveraging complex membership as a filter or scaffold in rare disease genomics could accelerate diagnosis and expand the catalogue of disease-linked epigenetic regulators.

Genome-wide DNA methylation profiling has become a valuable tool for enhancing diagnoses and assessing variant pathogenicity, particularly for epigenetic diseases. Variants in chromatin-modifying genes frequently disrupt methylation landscapes in a syndrome-specific manner, providing specific DNA methylation patterns for both diagnosis and mechanistic insight. These profiles can reflect underlying regulatory dysfunction and support variant classification, even when clinical features are subtle or do not fully match the expected syndrome (Aref-Eshghi et al., 2020; Trajkova et al., 2024). Importantly, methylation-based signatures are now recognized as robust molecular markers in many chromatin-related diseases and ongoing research continues to expand their applicability across a growing number of conditions (Sadikovic et al., 2020). As more of these signatures are discovered and validated, DNA methylation profiling is expected to play an increasingly central role in the diagnostic and classification workflow for rare neurodevelopmental diseases with an epigenetic basis.

In addition to advances in genomic and epigenomic diagnostics, another emerging approach involves the use of AI-based phenotyping platforms to support clinical interpretation. These tools are increasingly enabling integrative analysis of complex clinical manifestations by leveraging large-scale image, text and structured medical data (Dingemans et al., 2022; Hennocq et al., 2024). They are particularly valuable in the context of rare neurodevelopmental diseases involving epigenetic regulators which exhibit clinically well-recognizable facial phenotypes. By quantifying subtle craniofacial features and comparing them across reference cohorts, AI-driven phenotyping can support clinical suspicion, guide variant prioritization and refine diagnosis.

A major challenge in rare disease research extends beyond genomic variation to investigate epigenetic mechanisms: epigenetic deregulation is inherently context-dependent, varying across cell types, tissue environments and developmental stages. Therefore, detailed functional investigation requires access to relevant biological material or highly representative cellular models, which pose practical limitations in terms of expertise, cost and feasibility. Genetically engineered animal models and patient-derived cell lines provide valuable insightful information, but their generation is often resource-intensive requires a lot of resources. Recent advances in CRISPR/Cas9 genome editing and induced pluripotent stem cell (iPSC) technologies have enabled more precise modeling of patient-specific variants and chromatin dysregulation (Calzari et al., 2020; Fear et al., 2022; Foglia et al., 2024; Platt et al., 2017; Xiang et al., 2020). Furthermore, 3D cultures and organoid systems now offer more physiologically relevant platforms for studying human tissue architecture and epigenetic dynamics *in vitro* (Clevers, 2016; Dutta et al., 2017). In this context, model organisms such as mice, zebrafish, and fruit flies still provide essential insight into conserved chromatin regulatory pathways (Di Fede et al., 2022). Resources like MARRVEL, which integrate functional data from model organisms with human phenotype-genotype associations, are highly valuable for interpretation of rare or novel variants in epigenetic regulators (Wang et al., 2017).

As a result of growing understanding of epigenetic mechanisms in rare diseases, multidisciplinary efforts, like the Epigenetics and Chromatin Clinic at Johns Hopkins University, have emerged. These efforts integrate genomic, epigenomic and clinical data to provide comprehensive care (Harris et al., 2024). Such initiatives have potential to promote the shift toward precision medicine approaches in the diagnosis and management of epigenetic neurodevelopmental diseases.

## 6 Conclusion

Convergence in clinical and molecular features of diseases linked to histone modifications indicates the complex and dynamic nature of epigenetic regulation in neurodevelopment. The relationship between genotype and phenotype, which is shaped by factors such as the type of genetic variant, its location within specific domains and differences between individuals, emphasizes the need for comprehensive strategies to uncover shared disease mechanisms. As the boundaries between epigenetic pathways become less clear, the application of multi-omics tools, high-throughput chromatin mapping and AI-based network modeling offers significant potential for improving diagnostic precision and guiding therapy development. While genomic testing can detect genetic mutations, multi-omics and chromatin-focused methods are critical for understanding their functional consequences, revealing disruptions in regulation and explaining heterogeneity in phenotypical manifestations. Notably, some patients display clinical features resembling to known epigenetic syndromes but lack detectable mutations in potentially related chromatin regulators. These findings reveal the limitations in current genomic methods and raise the possibility that epigenetic problems may also arise from non-coding variants, changes in chromatin structure or post-translational modifications. Such insights emphasize the need for broad, system-level functional studies to fully capture the mechanisms in disease pathogenesis. In the future, integration of detailed clinical assessments with molecular network analysis will be essential for deepening our knowledge and identifying therapeutic targets for rare diseases that are genetically diverse but phenotypically overlapping.
