# Supplementary material for: Histone acetylation and methylation in rare diseases: from molecular mechanisms to clinical presentations
Source: Front Cell Dev Biol. 2026 Apr 9;14:1777625. doi: 10.3389/fcell.2026.1777625 (PMC13102643; doi:10.3389/fcell.2026.1777625)
Supplement: Supplementary file 1 [file Table1.docx]

**Supplementary table 1. Epigenetic functions and clinical implications of histone modifier genes**

| **Gene Function** | **Gene** | **Gene MIM Number** | **Histone** | **Related Syndrome** | **Phenotype MIM Number** | **Characteristic Manifestations** | **References** |
| --- | --- | --- | --- | --- | --- | --- | --- |
| **Lysine-specific methyltransferases (KMTs)** | *KMT2A* | 159555 | H3K4 | Wiedemann-Steiner syndrome (WDSTS) | 605130 | Thick eyebrows, long eyelashes, widely spaced eyes, narrow and downslanted palpebral fissures, hypertrichosis, hypotonia, DD/ID | (Sheppard and Quintero-Rivera, 1993) |
|  | *KMT2B* | 606834 | H3K4 | Dystonia 28, childhood-onset (DYT28)  Intellectual developmental disorder, autosomal dominant 68 | 617284  619934 | Lower-limb focal dystonia, generalized dystonia, communication difficulties, impaired swallowing, DD/ID, eye movement abnormalities, psychiatric comorbidities, seizures, spasticity, and sensorineural hearing loss  Short stature, poor overall growth, small head circumference, feeding difficulties, global developmental delay, impaired intellectual development, autism spectrum disorder, behavioral abnormalities | (Abela and Kurian, 1993) |
|  | *KMT2C* | 606833 | H3K4 | KMT2C-related syndrome  (previously known as Kleefstra syndrome 2) | 617768 | Poor growth, flattened midface, broad forehead, prominent eyebrows, short nose with anteverted nares, everted lower lip, DD/ID, autistic features, behavioral abnormalities | (Rots et al., 2024) |
|  | *KMT2D* | 602113 | H3K4 | Kabuki syndrome 1 | 147920 | Long palpebral fissures with eversion of the lateral third of the lower eyelid, arched and broad eyebrows, short columella with depressed nasal tip, prominent ears, persistence of fetal fingertip pads, mild-to-moderate ID, postnatal growth deficiency | (Adam and Hannibal, 1993) |
|  | *KMT2E* | 608444 | H3K4 | O’Donnell-Luria-Rodan syndrome (ODLURO) | 618512 | Mild-to-profound DD/ID, dolichocephaly, tall forehead, deep-set eyes, generalized hypotonia, seizures, autism spectrum disorder, sleep disturbance including frequent awakenings and difficulty falling asleep | (Pais et al., 1993) |
|  | *KMT2F* | 611052 | H3K4 | Early-onset epilepsy with or without developmental delay (EPEDD)  Neurodevelopmental disorder with speech impairment and dysmorphic facies (NEDSID) | 618832  19056 | Epilepsy, poor growth, cerebral dysplasia, white matter abnormalities  High forehead, full cheeks, microtia, hypertelorism, wide nose, wide mouth, everted upper lip, widely spaced teeth, feeding difficulties, joint hypermobility, GDD/ID, psychiatric abnormalities, behavioral problems | OMIM |
|  | *KMT2G* | 611055 | H3K4 | Intellectual developmental disorder with seizures and language delay (IDDSELD) | 619000 | Midface hypoplasia, full cheeks, thick lips, ear abnormalities, DD (especially speech and language delay), ID, seizures, autism spectrum disorder, behavioral abnormalities including hyperactivity, aggression and anxiety | OMIM |
|  | *KMT2H/ASH1L* | 607999 | H3K9,  H3K36 | Autosomal dominant intellectual developmental disorder-52 (MRD52) | 617796 | Variable dysmorphic facial features, feeding difficulties, gastrointestinal disturbances, hypotonia, autism spectrum disorder, DD/ID, poor or absent speech | OMIM |
|  | *KMT1D/EHMT1* | 607001 | H3K9 | Kleefstra syndrome 1 (KLEFS1) | 610253 | Brachycephaly, midface hypoplasia, coarse facies, hypertelorism, synophrys, everted lower lip, macroglossia, DD/ID, hypotonia, seizures, behavioral problems, sleep disorders | (Kleefstra and de Leeuw, 1993) |
|  | *EED* | 605984 | H3K27 | Cohen-Gibson syndrome (COGIS) | 617561 | Increased birth length and weight, excessive postnatal growth, macrocephaly, round face, broad forehead, large ears, hypertelorism, almond-shaped eyes, advanced bone age, osteopenia, scoliosis, large hands, long fingers, large feet, DD/ID, delayed speech | (Sequerra Amram Cohen and Gibson, 1993) |
|  | *EZH2* | 601573 | H3K27 | Weaver syndrome (WVS) | 277590 | Retrognathia, large and fleshy ears, round face, widely spaced eyes, almond-shaped palpebral fissures, tall stature, macrocephaly, variable ID, advanced bone age, poor coordination, soft and doughy skin, camptodactyly, umbilical hernia, abnormal tone, hypotonia | (Ocansey and Tatton-Brown, 1993) |
|  | *SUZ12* | 606245 | H3K27 | Imagawa-Matsumoto syndrome (IMMAS)/SUV12-related overgrowth | 618786 | Increased stature, increased weight, generalized postnatal overgrowth, macrocephaly, prominent forehead, round face, hypertelorism, downslanting palpebral fissures, advanced bone age, large hands and feet, hypotonia, DD/ID | OMIM |
|  | *KMT3A/SETD2* | 612778 | H3K36 | Luscan-Lumish syndrome (LLS) | 616831 | Macrocephaly, brain malformations, DD/ID, autism spectrum disorders, overgrowth or obesity, advanced bone age | (Pappas and Rabin, 1993) |
|  | *KMT3B/NSD1* | 606681 | H3K36 | Sotos Syndrome (SOTOS) | 117550 | Prominent forehead, dolichocephalic head shape, sparse frontotemporal hair, downslanting palpebral fissures, long and narrow face, DD/ID, overgrowth, autism spectrum disorder, advanced bone age, scoliosis | (Ocansey et al., 1993) |
|  | *KMT3G/NSD2* | 602952 | H3K36 | Rauch-Steindl syndrome (RAUST) | 619695 | Short stature, intrauterine and postnatal growth retardation, microcephaly, prominent forehead and glabella, triangular face, prominent ears, wide nasal bridge, DD/ID, autistic features, behavioral abnormalities | OMIM |
|  | *SETD5* | 615743 | H3K36 | Autosomal dominant intellectual developmental disorder-23 (MRD23) | 615761 | Brachycephaly, long and smooth philtrum, upslanting palpebral fissures, prominent and high nasal root, anteverted nares, DD/ID, kyphosis, scoliosis, obsessive-compulsive behavior, autistic features | OMIM |
|  | *KMT5B* | 610881 | H4K20 | Autosomal dominant intellectual developmental disorder-51 (MRD51) | 617788 | Macrocephaly, tendency toward tall stature, foot deformities, DD/ID, febril seizures, cryptorchidism, attention deficit problems, autism spectrum disorder | (Eliyahu et al., 2022) |
| **Lysine-specific demethylases (KDMs)** | *KDM1A* | 609132 | H3K4me1/2, H3K9me1/2 | Cleft palate, psychomotor retardation, and distinctive facial features (CPRF) | 616728 | Birth length and weight greater than 90%, brachycephaly, prominent forehead, frontal bossing, slightly arched eyebrows, downslanting palpebral fissures, wide nasal bridge, palatal abnormalities, tapered fingers, hypotonia, DD, speech delay, white matter hypoplasia | OMIM |
|  | *KDM3B* | 609373 | H3K9me1/2 | Diets-Jongmans syndrome | 618846 | Short stature, pointed chin, long ears, low hanging  columella, broad nasal tip, thin upper lip vermillion, wide mouth, DD/ID, speech delay, behavioral abnormalities | OMIM |
|  | *KDM4B* | 609765 | H3K9me2/3  H3K36me2/3 | Autosomal dominant intellectual  developmental disorder-65 (MRD65) | 619320 | Upslanting and narrow palpebral fissures, potruding metopic ridge, sparse temporal hair, depressed nasal bridge, microstomia, tented upper lip, feeding difficulties, short hands and feet, hypotonia, DD/ID, language delay, structural brain anomalies, clinodactyly | OMIM |
|  | *KDM5B* | 605393 | H3K4me1 | Autosomal recessive intellectual developmental disorder-65 (MRT65) | 618109 | Poor overall growth, dolichocephaly, square face, dysplastic ears, downslanting palpebral fissures, ptosis, high nasal bridge, bulbous nasal tip, thin lips, camptodactyly of the fourth and fifth fingers, DD/ID, speech delay, thin corpus callosum | OMIM |
|  | *KDM5C* | 314690 | H3K4me2/3 | Claes-Jensen type of X-linked syndromic intellectual developmental disorder (MRXSCJ) | 300534 | Small forehead, large ears, small and deep-set eyes, scrotal tongue, diastema, facial hypotonia, abundant body hair, short and thick distal phalanges, spasticity, epilepsy, short stature, obesity, behavioral disorders | OMIM |
|  | *KDM6A* | 300128 | H3K27me2/3 | Kabuki syndrome 2 | 300867 | Short stature, prominent ears, large auricle, sparse lateral eyebrows, long eyelashes, eversion of lateral third of lower eyelid, broad and/or depressed tip of nose, dental malocclusion, feeding difficulties in infancy, congenital heart defects, persistent fetal fingertip pads, DD/ID, speech delay, behavioral difficulties, hyperinsulinism | OMIM |
|  | *KDM6B* | 611577 | H3K27me2/3 | Neurodevelopmental disorder with coarse facies and mild distal skeletal abnormalities (NEDCFSA) | 618505 | Overgrowth, hypotonia, macrocephaly, prominent forehead, round face, full cheeks, strabismus, depressed nasal bridge, cleft lip/palate, joint hypermobility, poor feeding in infancy, hyperpigmented spots, DD/ID, spasticity, hypertonia, sleep disturbances | OMIM |
|  | *KDM7B/PHF8* | 300560 | H3K9me1/2 | Siderius-type X-linked syndromic intellectual developmental disorder (MRXSSD) | 300263 | Long face, sloping forehead, prominent supraorbital ridges, upslanting palpebral fissures, cleft lip/palate, long hands and toes, synophrys, dysarthric and unclear speech, DD/ID, nasal voice | OMIM |
| **Histone acetyltransferases (HATs)** | *CREBBP* | 600140 | H2A; H2B; H3 | Rubinstein-Taybi syndrome 1 (RSTS1)  Menke-Hennekam syndrome-1 (MKHK1) | 180849  618332 | Short stature, postnatal growth retardation, microcephaly, grimacing or unusual smile with almost closing of the eyes, heavy and high-arched eyebrows, downslanting palpebral fissures, beaked nose, small opening of the mouth, dental crowding, broad thumbs with radial angulation, broad great toes, DD/ID, severe expressive speech delay, seizures, recurrent infections  Short stature, microcephaly, prominent forehead, protruding ears, thick eyebrows, blepharophimosis, ptosis, broad nasal tip, short columella, recurrent upper airway infections, ulnar deviation of fingers, sparse hair, DD/ID, self-injurious behavior | OMIM, and (Stevens, 1993) |
|  | *EP300* | 602700 | H2A; H2B; H3 | Rubinstein-Taybi syndrome 2(RSTS2)  Menke-Hennekam syndrome-2 (MKHK2) | 613684  618333 | Microcephaly, micrognathia, heavy and high-arched eyebrows, beaked nose, dental malocclusion, broad thumbs, square distal fingertips, broad great toes, hypotonia, speech delay, DD/ID, hyperactivity  Prominent forehead, full cheeks, deep philtrum, short ears, hearing loss, upslanting palpebral fissures, blepharophimosis, delayed bone age, joint laxity, long fingers, broad halluces, DD/ID, autism, hyperactivity | OMIM, and (Stevens, 1993) |
|  | *KAT5 (TIP60)* | 601409 | H4; H2A | Neurodevelopmental disorder with dysmorphic facies, sleep disturbance, and brain abnormalities (NEDFASB) | 619103 | Small head circumference, round face, almond-shaped eyes, downturned corners of the mouth, dysphagia, fifth finger clinodactyly, scoliosis, poor or absent speech, DD/ID, seizures, sleep disturbances, abnormal corpus callosum, stereotypies, autism | OMIM |
|  | *KAT6A* | 601408 | H3K9,  H3K23,  H3K14 | Arboleda-Tham syndrome (ARTHS) | 616268 | Poor overall growth, microcephaly, plagiocephaly, microretrognathia, broad nasal tip, low-set ears, thin upper lip, downturned corners of the mouth, feeding difficulties, DD/ID, poor or absent speech, craniosynostosis | OMIM |
|  | *KAT6B* | 605880 | H3K9, H3K23 | Genitopatellar syndrome (GTPTS)  Say-Barber-Biesecker-Young-Simpson  syndrome (SBBYSS) | 606170  603736 | Microcephaly, coarse facies, downslanting palpebral fissures, hearing loss, pulmonary hypoplasia, hypoplasic scrotum, clitoral hypertrophy, cryptorchidism, multicystic kidneys, hip and limb contractures, absent patellae, dimple overlying knee, agenesis of corpus callosum, DD/ID  Microcephaly, prominent occiput, blepharophimosis, epicanthus inversus, orofacial cleft, structural cardiac defects, cryptorchidism, fifth-finger clinodactyly, long thumbs / great toes, hypoplastic teeth &/or delayed eruption of teeth, impaired speech, DD/ID | OMIM, and (Lemire et al., 1993) |
|  | *KANSL1* | 612452 | H4K16 | Koolen de Vreis syndrome (KDVS) | 610443 | Long face, high and broad forehead, large and prominent ears, upward-slanted palpebral fissures, blepharophimosis, epicanthal folds, pear-shaped nose, bulbous nasal tip, high and narrow palate, short stature, intrauterine growth retardation, heart defects, poor feeding, kidney/urologic anomalies, hypermobile joints, pigmentary abnormalities, hypotonia, DD/ID, autism, friendly behavior, nasal speech | OMIM, and (Koolen et al., 1993) |
|  | *KAT8* | 609912 | H4K16 | Li-Ghorgani-Weisz-Hubshman syndrome (LIGOWS) | 618974 | Low-set ears, hypotelorism, upslanted palpebral fissures, fullness of the upper lids, depressed nasal bridge, full lips, downturned corners of the mouth, fifth finger clinodactyly, brain imaging abnormalities, DD/ID, autism | OMIM |
| **Histone deacetylase (HDAC)** | *HDAC4* | 605314 |  | Neurodevelopmental disorder with central hypotonia and dysmorphic facies (NEDCHF) | 619797 | Delayed fontanel closure, frontal hair upsweep, large ears, hypertelorism, full lower lip, scoliosis, hip dislocation, DD/ID, hypotonia, sleep disturbance | OMIM |
|  | *HDAC6* | 300272 |  | X-linked dominant chondrodysplasia | 300863 | Short stature, intrauterine growth retardation, macrocephaly, frontal bossing, microphthalmia, short and flat nose, platyspondyly, rhizomelic shortening, metaphyseal cupping of phalanges, mild ID | OMIM |
|  | *HDAC8* | 300269 | H3/H4 | Cornelia de Lange syndrome-5 (CDLS5) | 300882 | Postnatal growth retardation, small head circumference, long philtrum, microretrognathia, arched eyebrows, synophrys, long eyelashes, depressed nasal bridge, downturned corners of the mouth, feeding problems, small hands and feet, nevus flammus, cutis marmorata, hypotonia, ID, behavioral disorders, happy demeanor | OMIM |

Abela, L., and Kurian, M. A. (1993). “KMT2B-Related disorders,” in *GeneReviews*®. Editors M. P. Adam, S. Bick, G. M. Mirzaa, R. A. Pagon, S. E. Wallace, and A. Amemiya (Seattle, WA: University of Washington, Seattle).

Adam, M. P., and Hannibal, M. (1993). “Kabuki syndrome,” in *GeneReviews*®. Editors M. P. Adam, S. Bick, G. M. Mirzaa, R. A. Pagon, S. E. Wallace, and A. Amemiya (Seattle, WA: University of Washington, Seattle).

Eliyahu, A., Barel, O., Greenbaum, L., Zaks Hoffer, G., Goldberg, Y., Raas-Rothschild, A., et al. (2022). Refining the phenotypic spectrum of KMT5B-Associated developmental delay. *Front. Pediatr.* 10, 844845. doi:10.3389/fped.2022.844845

Kleefstra, T., and de Leeuw, N. (1993). “Kleefstra syndrome,” in *GeneReviews*®. Editors M. P. Adam, S. Bick, G. M. Mirzaa, R. A. Pagon, S. E. Wallace, and A. Amemiya (Seattle, WA: University of Washington, Seattle).

Koolen, D. A., Morgan, A., and de Vries, B. B. (1993). “Koolen-de vries syndrome,” in *GeneReviews*®. Editors M. P. Adam, S. Bick, G. M. Mirzaa, R. A. Pagon, S. E. Wallace, and A. Amemiya (Seattle, WA: University of Washington, Seattle).

Lemire, G., Campeau, P. M., and Lee, B. H. (1993). “KAT6B disorders,” in *GeneReviews*®. Editors M. P. Adam, S. Bick, G. M. Mirzaa, R. A. Pagon, S. E. Wallace, and A. Amemiya (Seattle, WA: University of Washington, Seattle).

Ocansey, S., and Tatton-Brown, K. (1993). “EZH2-Related overgrowth,” in *GeneReviews*®. Editors M. P. Adam, S. Bick, G. M. Mirzaa, R. A. Pagon, S. E. Wallace, and A. Amemiya (Seattle, WA: University of Washington, Seattle).

Ocansey, S., Cole, T. R., Rahman, N., and Tatton-Brown, K. (1993). “Sotos syndrome,” in *GeneReviews*®. Editors M. P. Adam, S. Bick, G. M. Mirzaa, R. A. Pagon, S. E. Wallace, and A. Amemiya (Seattle, WA: University of Washington, Seattle).

Pais, L., Rodan, L., and O’Donnell-Luria, A. (1993). “KMT2E-Related neurodevelopmental disorder,” in *GeneReviews*®. Editors M. P. Adam, S. Bick, G. M. Mirzaa, R. A. Pagon, S. E. Wallace, and A. Amemiya (Seattle, WA: University of Washington, Seattle).

Pappas, J., and Rabin, R. (1993). “SETD2 neurodevelopmental disorders,” in *GeneReviews*®. Editors M. P. Adam, S. Bick, G. M. Mirzaa, R. A. Pagon, S. E. Wallace, and A. Amemiya (Seattle, WA: University of Washington, Seattle).

Sequerra Amram Cohen, A., and Gibson, W. T. (1993). “EED-related overgrowth,” in *GeneReviews*®. Editors M. P. Adam, S. Bick, G. M. Mirzaa, R. A. Pagon, S. E. Wallace, and A. Amemiya (Seattle, WA: University of Washington, Seattle).

Sheppard, S. E., and Quintero-Rivera, F. (1993). “Wiedemann-steiner syndrome,” in *GeneReviews*®. Editors M. P. Adam, S. Bick, G. M. Mirzaa, R. A. Pagon, S. E. Wallace, and A. Amemiya (Seattle, WA: University of Washington, Seattle).

Stevens, C. A. (1993). “Rubinstein-Taybi syndrome,” in *GeneReviews*®. Editors M. P. Adam, S. Bick, G. M. Mirzaa, R. A. Pagon, S. E. Wallace, and A. Amemiya (Seattle, WA: University of Washington, Seattle).
